# Predictors of seropositivity to SARS-CoV-2 among employees at a large urban medical center

**DOI:** 10.1186/s12889-024-20274-6

**Published:** 2024-10-09

**Authors:** Deborah Kupferwasser, Evelyn A. Flores, Prudencio Merino, Donna Phan Tran, Honghu Liu, Yilan Huang, Michael Bolaris, Megan H. Nguyen, Mildred Gonzales, Wellington Da Silva, Leslie Astorga-Cook, Angel Abueg, Holli Mason, Loren G. Miller

**Affiliations:** 1 Division of Infectious Diseases, The Lundquist Institute at Harbor-UCLA Medical Center, Torrance, CA USA; 2 Section of Public and Population Health, University of California, Los Angeles, Los Angeles, CA USA; 3 Department of Biostatistics, University of California, Los Angeles, Los Angeles, CA USA; 4 Department of Medicine, University of California, Los Angeles, Los Angeles, CA USA; 5 School of Pharmacy & Pharmaceutical Sciences, University of California, Irvine, CA USA; 6 Los Angeles County College of Nursing and Allied Health, Los Angeles, CA USA; 7 Los Angeles County + USC Medical Center, Los Angeles, CA USA; 8 Lundquist Institute of Biomedical Innovation at Harbor-UCLA Medical Center, United States, 1124 W. Carson St, Torrance, CA 90502 USA

**Keywords:** SARS-CoV-2, Seropositivity, Health system workforce

## Abstract

**Background:**

Before SARS-CoV-2 vaccination availability, medical center employees were at high risk of COVID-19. However, risk factors for SARS-CoV-2 infection in medical center employees, both healthcare and non-healthcare workers, are poorly understood.

**Methods:**

From September-December 2020, free IgG antibody testing was offered to all employees at a large urban medical center. Participants were asked to complete a questionnaire on work and non-work related risk factors for COVID-19 infection.

**Results:**

SARS-CoV-2 seropositivity was found in 4.7%. Seropositivity was associated with close contact with COVID-19 cases with or without the use of adequate personal protective equipment (PPE), (OR 3.1 [95% CI 1.4–6.9] and OR 4.7 [95% CI 2.0–11.0] respectively), never wearing a mask outside of work (OR 10.1 [95% CI 1.9–57]), and Native Hawaiian/Pacific Islander race (OR 6.3 95% CI (1.6–25)].

**Conclusions:**

Among workers in a large urban medical center, SARS-CoV-2 seropositivity was associated with work-related COVID-19 close contacts and low mask use outside of work, suggesting that non-workplace close contacts are also relevant routes of COVID-19 spread among healthcare workers.

**Supplementary Information:**

The online version contains supplementary material available at 10.1186/s12889-024-20274-6.

## Background

The SARS-CoV-2 pandemic has caused significant loss of life, health, and economic output. As of November 2023, COVID-19 cases obtained by the World Health Organization show 772 million confirmed cases and 6 million deaths worldwide [1]. During the early months of the SARS-CoV-2 pandemic, healthcare systems experienced an amplified demand for services due to increasing COVID-19 cases. The increase in demand for healthcare services strained healthcare workforces, who were at a higher risk of exposure to SARS-CoV-2, subsequent infection, and absenteeism [2, 3].

The occupational burden of the SARS-CoV-2 pandemic has been substantial. In California, 73% of COVID-19 cases and 29% of COVID-19 deaths have been in working-age (18–64 years) adults [4]. In addition, minority populations were disproportionally affected by the SARS-CoV-2 pandemic, experiencing higher COVID-19 infection rates and mortality [5, 6]. Furthermore, a nationwide United States study showed minority populations were more likely to be employed in occupations with potentially higher SARS-CoV-2 exposure risks, and were less likely to have options to work from home [6]. These factors may have been important drivers of the health disparity seen in COVID-19 infections in the U.S [6].

Frontline healthcare workers are at a higher risk of testing positive for the SARS-CoV-2 virus than the general population [2]. Moreover, data from the California Division of Occupational Safety and Health (Cal/OSHA) show that healthcare was the most common industry for COVID-19 workers’ compensation claims [7]. Before the availability of the SARS-CoV-2 vaccine, global seroprevalence in the general population was found to be 4.5% [8]. In the pre-SARS-CoV-2 vaccine era, the seroprevalence among healthcare workers was 8%, demonstrating that healthcare workers are at higher risk for COVID-19 infection [9].

Although several studies have assessed risks of SARS-CoV-2 in healthcare systems, a comprehensive understanding of the occupational risk of SARS-CoV-2 exposure that includes behaviors, opinions and beliefs about SARS-CoV-2 exposure risk is incomplete [2, 10, 11]. To address this lack of knowledge, we analyzed data from a SARS-CoV-2 IgG seroprevalence program among Los Angeles County + University of Southern California (LAC + USC) Medical Center (currently named Los Angeles General Medical Center) employees, both healthcare workers and non-healthcare workers. We hypothesized that seroprevalence of SARS-CoV-2 IgG antibodies will be higher in LAC + USC employees who participated in direct patient care activities compared to those who had no direct patient care activities. Our program aimed to identify risk factors for SARS-CoV-2 IgG antibodies, including attitudes and beliefs related to the potential COVID-19 vaccine. Our program sample population included a racially and ethnically diverse population with varying levels of occupational risk.

## Methods

### Program design, setting, and population

We analyzed data from a seroprevalence program conducted in our health system that occurred from September 2020-December 2020, prior to the availability of the COVID-19 vaccine. Recruitment of program participants occurred at LAC + USC, where employees were notified via repeated email blasts about the voluntary SARS-CoV-2 IgG serosurvey program. All LAC + USC adult employees were eligible to participate in the program. Program personnel were stationed outside the LAC + USC medical center entrance where interested persons could approach and be screened for eligibility via a short interview to confirm LAC + USC employment.

### Serology

SARS-CoV-2 IgG serology was completed using Abbott Laboratories SARS-CoV-2 IgG nucleocapsid protein ELISA kit™ (Abbott Diagnostics, IL, USA). Antibody immunoassay was performed as previously described [12]. Samples were considered positive if antibody levels were ≥ 1.4 (manufacturer’s arbitrary units as described in the product use information sheet) [13]. The sensitivity and specificity for the detection of SARS-CoV-2 IgG nucleocapsid protein has been reported to be approximately 99% and 99% respectively [12]. Blood sampling procedures were performed by students from the Los Angeles County College of Nursing and Allied Health. Specimens were processed on the Abbott Architect system in a Clinical Laboratory Improvement Amendments certified clinical laboratory.

### Program questionnaire

All program participants were asked to complete a questionnaire at the time of blood sampling. The program questionnaire could be accessed electronically on participants cell phones via a QR code that was available during check in for the serology program. The QR code led to a Survey Monkey-based questionnaire (Survey Monkey^®^, San Mateo, California USA). Alternatively, if participants could not or would not do the survey on their phone, a paper version was offered.

The questionnaire was developed to collect information on employees’ risk of COVID-19 exposure both at home and at work along with attitudes and beliefs towards the potential COVID-19 vaccine. Exposure was defined as coming in close contact, less than 6 feet from another person. The questionnaire consisted of a total of 44 questions divided into seven sections: **1.** Possible prior COVID-19 exposures; **2**. Possible prior COVID-19 exposures working in the LAC + USC healthcare system during patient and/or coworker interactions; these exposures were subcategorized into exposures when adhering to infection prevention measures (i.e., social distancing, masking and hand-hygiene) or when not adhering to these measurers; **3**. Possible prior COVID-19 exposures during non-LAC + USC healthcare working hours but other institutional work, such as volunteer work, or part-time work (i.e., following infection prevention measures described above); **4**. Possible prior COVID-19 exposures in the household and other non-work settings; **5**. Beliefs and attitudes towards a potential COVID-19 vaccine, including reasons why or why not participants would get vaccinated with the COVID-19 vaccine when it became available; **6**. Demographics; **7**. Opinions about a potential COVID-19 vaccine; this section used items derived using the Health Belief Model constructs of perceived susceptibility, threat, and self-efficacy [14]. Additionally, questions comprising the Health Belief Model constructs of perceived susceptibility, threat, and self-efficacy were previously evaluated for internal consistency by calculating Cronbach’s alpha [15]. Although our program questionnaire is not a validated survey instrument, it incorporates response items relevant to the COVID-19 pandemic as reported by other studies conducted on similar populations [10, 16, 17].

In our questionnaire (Supplemental Table 1), questions 1 A-1Bii ask about COVID-19 exposure, incorporating survey items from a published study [2]. We modified the survey items to make them specific for our setting, for example by including the name of our system, the Department of Health Services (DHS), and added items on Personal Protective Equipment (PPE) use during a possible exposure. Questions 2 A-2 J were developed for this investigation and query about patient contact and number of hours worked at DHS facilities. Questions 3 A-3 F and 4 A-4D were also developed for this investigation and query about non-DHS employment, volunteer activities and possible exposures with these activities. Of note, questions in Sects. 2–4 were developed by a working group that consisted of experts in the field of Infectious Disease and hospital infection control practices (co-authors: LGM, MB, and MHN) and underwent pilot testing and revision for clarify, accuracy, and validity. Questions 5 A-5D were based on a published study [18] and queried about attitudes toward a COVID-19 vaccine. Questions 6 A-6E were demographic questions and questions 7 A-7 J were survey items on phycological constructs on attitudes, beliefs, and self-efficacy. Items were based on the Health Belief Model [19, 20] and items were tested and revised for clarify, accuracy, and validity. All response items included check boxes, fill-in, or 7-point Likert type questions that ranged from strongly disagree, disagree, somewhat disagree, neither agree or disagree, somewhat agree, agree, to strongly agree.

### Statistical methods

To determine the work-related and non-work-related risk factors for seropositivity, bivariate, and logistic regression were performed. Bivariate analyses involved examining the marginal relationship between each risk factor and the outcome variable independently, and logistic regression was used to model the binary outcome of seropositivity. The Cochran-Armitage test was used to test for a trend in the proportion of the seropositivity across categories of an ordinal variable, such as COVID-19 exposure level at work. For continuous variables, such as age, marginal logistic regression was applied to model the relationship between the continuous variable and the outcome. Predictor variables that showed a significant association with seropositivity at *P*-value < 0.2 were included in the final multivariable logistic regression model. All analysis were performed using SAS software version 9.4 (SAS Institute^®^, Cary NC).

### Ethical approval and consent to participate

No personal identifiers or protected health information was collected for the program. Human ethics and consent to participate declarations are not applicable as the program was determined to not be human subject research as defined by Department of Health and Human Services and or Federal Drug Administration regulations. Review of the program protocol was conducted by the following institutional review board (IRB) ethics committee, The John F. Wolf M.D Human Subject Committee. The need of informed consent was waved as the project was determined to not be human subject research as detailed above. The employee serology program was originally intended to be run at all hospitals in the Los Angeles County DHS system, a safety net healthcare system that includes LAC + USC and Harbor-UCLA, although for logistic reasons (phlebotomist shortage) it was only offered at the former medical center. This work was supported by an investigator-initiated grant from Merck which had no role in the study design, data collection, interpretation of findings, or manuscript writing.

## Results

Serology and administration of the program questionnaire occurred from September 1st to December 31st, 2020. Of the approximately 10,500 persons who were eligible for this program, 1,327 participated. Among program participants, 1,273 completed the questionnaire and were included in the analysis. (Table 1) Seropositivity for SARS-CoV-2 IgG antibodies was 60/1,273 (4.7%). (Table 1)

**Table 1:** Demographics, exposure, and behaviors: Univariate and Bivariate Analysis

	*Total N*	Seropositive *N (%)*	Seronegative *N (%)*	*OR*	*95% CI*	*p-value*
	1273	60 (4.7)	1213 (95)			
**Age in years** mean (SD)	35 (17)	32 (10)	38 (14)	0.98	0.97–1.0	0.03
**Race/ethnicity**						
Asian or Asian American	354 (28)	12 (20)	342 (28)	REF	-	-
Black or African American	70 (6)	3 (5.0)	67 (5.5)	1.3	0.35–4.7	0.71
White or Caucasian	242 (19)	9 (15)	233 (19)	1.1	0.46–2.7	0.83
Hispanic/Latino	449 (35)	26 (43)	423 (35)	1.8	0.87–3.5	0.12
Native Hawaiian or other Pacific Islander	26 (2.0)	3 (5.0)	23 (1.9)	3.7	0.91–14	0.05
Other, Decline to state, American Indian or Alaska Native, Mixed Race	132 (10)	7 (12)	125 (10)	0.46	0.06–3.6	0.46
**Gender**						
Male	369 (29)	18 (30)	351 (27)	REF	—	—
Female	857 (67)	39 (65)	818 (67)	0.93	0.53–1.7	0.99
**Employment Type**						
Physician	228 (17)	6 (10)	222 (17)	0.76	0.23–2.5	0.65
Nurse	556 (44)	36 (60)	520 (41)	1.8	0.69–4.7	0.23
Non-patient based role	206 (16)	6 (10)	200 (16)	0.93	0.31–2.8	0.91
Patient-based care, non-physician, non-nurse	159 (12)	6 (10)	153 (12)	REF	—	—
**Prior known COVID-19 exposure**						
No exposure	484 (38)	9 (15)	475 (39)	REF	-	-
Exposure w/out PPE	244 (19)	20 (33)	224 (19)	5.3	2.3–12	< 0.0001
Exposure w/PPE	542 (43)	31 (52)	511 (42)	3.6	1.6–7.9	0.001
**Did you have or think you had a prior COVID-19 infection**						
No	957 (75)	10 (17)	947 (78)	REF	-	-
Yes, and one or more SARS-CoV-2 tests were positive	61 (5)	40 (67)	21 (2)	176	77–399	< 0.0001
Yes, and all SARS-CoV-2 tests were negative	130 (10)	5 (8)	125 (10)	3.9	1.3–11	0.01
Yes, but did not get a SARS-CoV-2 test	122 (10)	5 (8)	117 (10)	4.2	1.4–12	0.01
**Hours worked in the medical center per week**						
Mean (SD)	38 (15)	36 (20)	39 (15)	0.99	0.9-1.0	0.27
**Characteristics of work conditions as it relates to patient exposure**						
Number of physical contacts with patients per **day**^**a**^						
Never	191 (15)	5 (9)	186 (16)	1.3	1.1–1.6	0.01
Rarely (1–10 physical contacts/day)	299 (24)	7 (12)	292 (24)			
Occasionally (11–20 physical contacts/day)	228 (18)	13 (23)	215 (18)			
Frequently (21–30 physical contacts/day)	212 (17)	10 (18)	202 (17)			
Very frequently (> 30 physical contacts/day)	323 (26)	22 (39)	301 (25)			
Number of physical contacts with known COVID-19-infected patients per **day**^**a**^						
Never	445 (36)	13 (23)	432 (36)	1.4	1.1–1.7	0.003
Rarely (1–10 physical contacts/day)	487 (39)	21 (37)	466 (39)			
Occasionally (11–20 physical contacts/day)	188 (15)	12 (21)	176 (15)			
Frequently (21–30 physical contacts/day)	75 (6)	5 (9)	70 (6)			
Very frequently (> 30 physical contacts/day)	58 (5)	6 (11)	52 (4)			
Percent of **time**^**b**^ spent within an area where patients come within 6 feet of you						
Never	125 (10)	2 (4)	123 (10)	1.2	0.94–1.4	0.17
Rarely (1–25% of the time)	238 (19)	11 (19)	227 (19)			
Occasionally (26–50% of the time)	194 (15)	6 (11)	188 (16)			
Frequently (51–99% of the time)	253 (20)	17 (30)	236 (20)			
Always (100% of the time)	445 (35)	21 (37)	424 (35)			
Percent of **time**^**b**^ spent within an area where known COVID-19-infected patients come within 6 feet of you						
Never	367 (29)	12 (21)	355 (30)	1.2	1.0-1.5	0.054
Rarely (1–25% of the time)	412 (33)	16 (28)	396 (33)			
Occasionally (26–50% of the time)	221 (18)	13 (23)	208 (17)			
Frequently (51–99% of the time)	118 (9)	8 (14)	110 (9)			
Always (100% of the time)	135 (11)	8 (14)	127 (11)			
**Characteristics of work conditions as it relates to mask use while working**						
Percent of **time**^**b**^ spent wearing a mask while working within 6 feet of patients						
Never	16 (1)	0 (0)	16 (1)	1.3	0.80–2.1	0.33
Rarely (1–25% of the time)	20 (2)	0 (0)	20 (2)			
Occasionally (26–50% of the time)	13 (1)	0 (0)	13 (1)			
Frequently (51–99% of the time)	73 (6)	4 (7)	69 (6)			
Always (100% of the time)	1082 (86)	52 (91)	1030 (86)			
I don’t interact with patients and never come within 6 feet of patients	49 (4)	1 (2)	48 (4)			
Number of physical contacts with co-workers per **day**^**a**^						
Never	132 (11)	7 (12)	125 (10)	1.1	0.90–1.3	0.43
Rarely (1–10 physical contacts/day)	308 (25)	13 (23)	295 (25)			
Occasionally (11–20 physical contacts/day)	176 (14)	3 (5)	173 (15)			
Frequently (21–30 physical contacts/day)	219 (18)	11 (19)	208 (17)			
Very frequently (> 30 physical contacts/day)	414 (33)	23 (40)	391 (33)			
Percent of **time**^**b**^ spent within an area where co-workers come within 6 feet of you						
Never	16 (1)	1 (2)	15 (1)	0.92	0.72–1.2	0.50
Rarely (1–25% of the time)	115 (9)	6 (11)	109 (9)			
Occasionally (26–50% of the time)	174 (14)	10 (18)	164 (14)			
Frequently (51–99% of the time)	377 (30)	15 (26)	362 (30)			
Always (100% of the time)	567 (45)	25 (44)	542 (45)			
Percent of **time**^**b**^ spent wearing a mask where co-workers come within 6 feet of you						
Never	11 (1)	1 (2)	10 (1)	1.6	0.82–2.9	0.07
Rarely (1–25% of the time)	22 (2)	0 (0)	22 (2)			
Occasionally (26–50% of the time)	40 (3)	0 (0)	40 (3)			
Frequently (51–99% of the time)	374 (30)	12 (21)	362 (30)			
Always (100% of the time)	802 (64)	44 (77)	758 (64)			
Percent of **time**^**b**^ spent practicing adequate hand hygiene						
Never	6 (1)	0 (0)	6 (1)	1.5	0.82–2.9	0.18
Rarely (1–25% of the time)	10 (1)	0 (0)	10 (1)			
Occasionally (26–50% of the time)	17 (1)	0 (0)	17 (1)			
Frequently (51–99% of the time)	232 (19)	9 (16)	223 (19)			
Always (100% of the time)	984 (79)	48 (84)	936 (79)			
**COVID-19 exposure outside medical center**						
Number of additional persons living in the home						
0	147 (12)	9 (16)	138 (12)	REF	-	-
1 or more	1095 (88)	49 (84)	1046 (88)	0.72	0.4–1.5	0.38
Age group living in the home						
Live Alone	147 (12)	9 (16)	138 (12)	REF	—	—
Number of persons who live in home that are child age 0–5, mean (median)	0.99 (1)	1.1 (1)	0.97 (1)	1.6	0.43–5.8	0.50
Number of persons who live in home that are child age 6–12, mean (median)	1.1 (1)	1.3 (1)	1.1 (1)	0.4	0.02–7.2	0.51
Number of persons who live in home that are child Age 13–17, mean (median)	0.91 (1)	0.86 (1)	0.91 (1)	0.6	0.03-13	0.77
Number of persons who live in home that are adult, mean (median)	2 (2)	1.8 (1)	2 (2)	1.0	0.47–2.1	0.931
Number of persons who live in home that are either children or adults mean (median)	4.8 (5)	4.5 (5)	4.8 (5)	0.4	0.18–0.97	0.043
Number of people in the home, mean (median)	2.7 (1)	2.3 (2)	2.7 (2)	0.8	0.6-1.0	0.09
**Number**^**c**^ of trips outside home (shopping or errands)						
Never	30 (2)	1 (2)	29 (2)	1.5	0.86–2.5	0.1664
1–2 times/week	992 (80)	45 (78)	947 (80)			
3–6 times a week	206 (17)	9 (16)	197 (17)			
7 times a week or more	12 (1)	3 (5)	9 (1)			
Percent of **time**^**b**^ wearing a mask outside the home (excluding time wearing a mask at work)						
Never	22 (2)	2 (3)	20 (2)	0.79	0.60-1.0	0.0791
Rarely (1–25% of the time)	25 (2)	1 (2)	24 (2)			
Occasionally (26–50% of the time)	40 (3)	4 (7)	36 (3)			
Frequently (51–99% of the time)	216 (17)	13 (22)	203 (17)			
Always (100% of the time)	936 (76)	38 (66)	898 (76)			
Percent of **time**^**b**^ practicing hand hygiene (excluding time while at work)						
Never	10 (1)	0 (0)	10 (1)	1.3	0.86-2.0	0.2181
Rarely (1–25% of the time)	9 (1)	1 (2)	8 (1)			
Occasionally (26–50% of the time)	69 (6)	2 (3)	67 (6)			
Frequently (51–99% of the time)	509 (41)	20 (34)	489 (41)			
Always (100% of the time)	641 (52)	35 (60)	606 (51)			
Frequency of getting the annual Flu (influenza) vaccine						
Never	48 (4)	3 (5)	45 (4)	REF	—	—
Sometimes	125 (10)	6 (11)	119 (10)	0.76	0.18–3.2	0.70
Always	1061 (86)	48 (84)	1013 (86)	0.71	0.21–2.4	0.58

^**a**^ **Scale for the number of physical contacts per** ***day***

1 = Never, 2 = Rarely (1–10 physical contacts/day), 3 = Occasionally (11–20 physical, contacts/day), 4 = Frequently (21–30 physical contacts/day), 5 = Very frequently (> 30 physical contacts/day)

^**b**^ **Scale for the percent of** ***time***

1 = Never, 2 = Rarely (1–25% of the time), 3 = Occasionally (26–50% of the time), 4 = Frequently (51–99% of the time), 5 = Always (100% of the time)

^**c**^ **Scale for the** ***number*** **of trips outside the home**

1 = Never, 2 = 1–2 times/week, 3 = 3–6 times a week, 4 = 7 times a week or more

Not all program participants responded to all questions, so totals may not add up to 1273. Scales for response items that were converted to continuous values for univariate and bivariate analyses are as follows:

Mean participant age was 35 (S.D: 16.9), with a range of 25–79 years. (Table 1) The most common race/ethnicity of participants was Hispanic/Latino (449, 37%), followed by Asian or Asian American (354, 29%), Caucasian (242, 20%), Black African American (70, 5.8%), Native Hawaiian/Pacific Islander (NHPI) (26, 2.2%), and “other” race/ethnicity (63, 5.2%) (Table 1). Gender distribution was 857 (67%) females and 369 (29%) males. (Table 1) The most common employment category among participants were nurses (556, 44%), followed by physicians (228, 17%), employees with non-patient-based roles (206, 16%), and allied health professionals (i.e., patient-based care, non-physician, non-nurse healthcare workers such as physical, occupational, and respiratory therapists) (159, 12%). (Table 1) The mean weekly hours worked in the medical center was 38 (SD 15.4). (Table 1)

The majority of participants indicated they did not have a known previous COVID-19 infection, (957, 75%). (Table 1) Three hundred and thirteen (25%) program participants indicated they had or think they had a prior COVID-19 infection. (Table 1) Among the participants who had or thought they had a prior COVID-19 infection, 61 (5%) indicated they got tested for SARS-CoV-2 with one or more tests being positive, 130 (10%) indicated they were tested but the results were negative, and 122 (10%) indicated they did not get a test. (Table 1) Among program participants, 542 (43%) reported COVID-19 exposure while wearing PPE, 244 (19%) reported COVID-19 exposure while not wearing PPE and 484 (38%) reported no COVID-19 exposure. (Table 1) The majority of participants indicated that they always receive the annual influenza vaccine, (1061, 86%). (Table 1)

Details of program participants patient contact frequency and percent of time spent within 6 feet of patients are outlined in Table 1. Hospital infection control measures were followed by the majority of program participants. Participants indicated that they always (100% of the time) wear a mask when coming within 6 feet of patients, (1082, 86%). (Table 1) Additionally, the majority (984, 79%) of participants indicated they always (100% of the time) practice hand hygiene while at work. (Table 1)

The results from questions characterizing working conditions as they relate to co-workers show that 414 (33%) participants indicated very frequent (> 30 physical contacts/day) with co-workers. Additionally, 567 (45%) participants indicated that they were always (100% of the time) within 6 feet of co-workers, and 802 (64%) said they always (100% of the time) wore a mask when co-workers were within 6 feet from them at work. (Table 1)

The majority of participants lived with one or more persons (1095, 88%). (Table 1) The median number of household children age 0–5 years, age 6–12, and age 13–17 was 1 for each age category. The median number of household adults (not including yourself) was 2 and the median number of household members in participants’ residence was 5. (Table 1)

In terms of COVID-19 exposure outside working hours, we found that the majority of the participants went outside the house 1–2 times/week, (992, 80%). (Table 1) Most (936, 76%) participants said that when they were outside, they always (100% of the time) wear a mask. (Table 1) Excluding while at work, respondents reported they always (641, 52%) or frequently (509, 41%) practiced hand hygiene. (Table 1)

Attitudes and beliefs about the potential COVID-19 vaccines among program participates are displayed in Table 2. Responses to the questions, “If I am not careful, I will get COVID infection at work” and “If I am not careful, I will get COVID infection while in the community doing activities such as shopping, and interacting with others”, was strongly agree (518, 42%) and, (518, 42%), respectively. Distributions of responses to other questions related to attitudes and beliefs are summarized in Table 2.

**Table 2:** Attitudes and beliefs about COVID-19 vaccine: Univariate and Bivariate Analysis

	*Total N*	Seropositive *N (%)*	Seronegative *N (%)*	*OR*	*95% CI*	*p-value*
Do you plan to get the COVID vaccine when available?						
No/probably no	267 (22)	17 (30)	250 (21)	REF		
Yes/probably yes	968 (78)	40 (70)	928 (79)	0.63	0.35 − 0.14	0.13
I am worried about being infected by COVID						
Strongly disagree	46 (4)	1 (2)	45 (4)	0.92	0.78–1.08	0.28
Disagree	51 (4)	6 (11)	45 (4)			
Somewhat disagree	43 (4)	4 (7)	39 (3)			
Neither agree nor disagree	145 (12)	7 (12)	138 (12)			
Somewhat agree	253 (21)	6 (11)	247 (21)			
Agree	366 (30)	21 (37)	345 (30)			
Strongly agree	320 (26)	12 (21)	308 (26)			
COVID affects me emotionally						
Strongly disagree	89 (7)	1 (2)	88 (8)	1.00	0.86–1.2	0.93
Disagree	160 (13)	12 (21)	148 (13)			
Somewhat disagree	72 (6)	4 (7)	68 (6)			
Neither agree nor disagree	215 (18)	10 (18)	205 (18)			
Somewhat agree	344 (28)	13 (23)	331 (28)			
Agree	225 (18)	9 (16)	216 (19)			
Strongly agree	119 (10)	8 (14)	111 (10)			
Wearing a mask is important to protect me from COVID						
Strongly disagree	30 (2)	1 (2)	29 (2)	1.0	0.80–1.2	0.93
Disagree	7 (1)	0 (0)	7 (1)			
Somewhat disagree	18 (1)	1 (2)	17 (1)			
Neither agree nor disagree	41 (3)	4 (7)	37 (3)			
Somewhat agree	53 (4)	1 (2)	52 (4)			
Agree	219 (18)	11 (19)	208 (18)			
Strongly agree	856 (70)	39 (68)	817 (70)			
Frequent hand hygiene is important to protect me from getting COVID						
Strongly disagree	26 (2)	1 (2)	25 (2)	1.1	0.79–1.4	0.71
Disagree	3 (0)	0 (0)	3 (0)			
Somewhat disagree	1 (0)	0 (0)	1 (0)			
Neither agree nor disagree	13 (1)	0 (0)	13 (1)			
Somewhat agree	12 (1)	1 (2)	11 (1)			
Agree	185 (15)	9 (16)	176 (15)			
Strongly agree	983 (80)	46 (81)	937 (80)			
Outside of my home, keeping more than 6 feet away from others is important						
Strongly disagree	24 (2)	1 (2)	23 (2)	1.1	0.84–1.4	0.56
Disagree	11 (1)	0 (0)	11 (1)			
Somewhat disagree	8 (1)	0 (0)	8 (1)			
Neither agree nor disagree	32 (3)	1 (2)	31 (3)			
Somewhat agree	71 (6)	6 (11)	65 (6)			
Agree	304 (25)	10 (18)	294 (25)			
Strongly agree	774 (63)	39 (68)	735 (63)			
It is easy for me to do things at work and home to prevent me from getting COVID infection						
Strongly disagree	24 (2)	0 (0)	24 (2)	1.2	0.10–1.5	0.14
Disagree	32 (3)	1 (2)	31 (3)			
Somewhat disagree	69 (6)	1 (2)	68 (6)			
Neither agree nor disagree	95 (8)	4 (7)	91 (8)			
Somewhat agree	228 (19)	11 (19)	217 (19)			
Agree	390 (32)	21 (37)	369 (32)			
Strongly agree	384 (31)	19 (33)	365 (31)			
If I am not careful, I will get COVID infection at work						
Strongly disagree	19 (2)	1 (2)	18 (2)	1.2	0.96–1.6	0.10
Disagree	17 (1)	1 (2)	16 (1)			
Somewhat disagree	15 (1)	0 (0)	15 (1)			
Neither agree nor disagree	87 (7)	3 (5)	84 (7)			
Somewhat agree	186 (15)	5 (9)	181 (16)			
Agree	381 (31)	14 (25)	367 (31)			
Strongly agree	518 (42)	33 (58)	485 (42)			
If I am not careful, I will get COVID infection while in the community doing activities such as shopping, and interacting with others						
Strongly disagree	21 (2)	1 (2)	20 (2)	1.4	1.1–1.9	0.02
Disagree	13 (1)	0 (0)	13 (1)			
Somewhat disagree	23 (2)	2 (4)	21 (2)			
Neither agree nor disagree	64 (5)	2 (4)	62 (5)			
Somewhat agree	197 (16)	3 (5)	194 (17)			
Agree	387 (32)	11 (19)	376 (32)			
Strongly agree	518 (42)	38 (67)	480 (41)			
If I get COVID infection, I will get severely ill and may need to be hospitalized						
Strongly disagree	21 (2)	1 (2)	20 (2)	1.0	0.90–1.2	0.69
Disagree	104 (9)	6 (11)	98 (8)			
Somewhat disagree	93 (8)	5 (9)	88 (8)			
Neither agree nor disagree	383 (31)	19 (33)	364 (31)			
Somewhat agree	234 (19)	2 (4)	232 (20)			
Agree	226 (18)	12 (21)	214 (18)			
Strongly agree	161 (13)	12 (21)	149 (13)			
People around me at work at the medical center are routinely wearing masks when they are less than 6 feet away from me and others						
Strongly disagree	22 (2)	2 (4)	20 (2)	0.91	0.80–1.10	0.34
Disagree	18 (1)	2 (4)	16 (1)			
Somewhat disagree	35 (3)	1 (2)	34 (3)			
Neither agree nor disagree	37 (3)	1 (2)	36 (3)			
Somewhat agree	150 (12)	9 (16)	141 (12)			
Agree	473 (39)	20 (35)	453 (39)			
Strongly agree	487 (40)	22 (39)	465 (40)			

Likert-type 7-point response item scale

1 = Strongly disagree, 2 = Disagree, 3 = Somewhat disagree, 4 = Neither agree or disagree, 5 = Somewhat agree, 6 = Agree, 7 = Strongly agree

Not all program participants responded to all questions, so totals may not add up to 1273. Scales for response items that were converted to continuous values for univariate and bivariate analyses are as follows:

### Bivariate analysis

Among demographic variables, in bivariate analysis, SARS-CoV-2 seropositivity was associated with participants’ age (OR 0.98 [95% CI 0.97–1.00]), but not gender, race, or employment type. (Table 1) In terms of program participants prior COVID-19 infection, seropositivity was associated with reporting a previously positive test for SARS-CoV-2 (OR 175.8 [95% CI 77.6-398.6]), reporting likely prior COVID-19 infection but testing negative for SARS-CoV-2 antibody (OR 3.9 [95% CI 1.3–11.4]), and participants who thought they had prior COVID-19 but did not get a SARS-CoV-2 antibody test at the time of symptoms (OR 4.2 [95% CI 1.4–12.5]). (Table 1)

In terms of prior SARS-CoV-2 exposure, seropositivity was associated with COVID-19 exposure with PPE use (OR 3.61 [95% CI 1.64–7.93)] and COVID-19 exposure without PPE use (OR 5.31[ 95% CI 2.30-12.22)]. (Table 1) In terms of variables that characterize participants’ work, for every increase in unit of frequency of physical contacts with patient the odds of seropositivity increased 31% (OR 1.31 [95% CI 1.08–1.60)]. (Table 1) Also, for every increase in unit of frequency of physical contacts with hospitalized COVID-19 patients, the odds of seropositivity increased 39% (OR 1.39 [95% CI 1.12–1.72)]. (Table 1) Seropositivity was inversely associated with exposure to living with either children or adults. The odds of seropositivity decreases 4% for every additional person (either child or adult) living with a program participant (OR 0.04 [95% CI 0.18–0.97)]. (Table 1) In terms of program participants’ attitude and opinion about the potential COVID-19 vaccine, seropositivity was associated with strongly agreeing to the statement “If I am not careful, I will get COVID infection while in the community doing activities such as shopping, and interacting with others” (OR 1.41 [95% CI 1.06–1.89)]. (Table 2)

### Multivariate analysis

In our multivariate model, independent predictors of seropositivity included SARS-CoV-2 exposure at work using personal protective equipment (PPE), (OR 3.1 [95% CI 1.4–6.9]) and SARS-CoV-2 exposure at work without using PPE, (OR 4.7 [95% CI (2.0–11.0)] (referent group: no COVID-19 exposure at work). (Table 3) Additionally, seropositivity was associated with participants who indicated never wearing a mask outside of work compared to participants who frequently/always wear a mask outside of work (OR 10.3 [95% CI 1.9–57)]. (Table 3) Seropositivity was also associated with identifying as NHPI race/ethnicity (OR 6.3 [95% CI 1.6–25], referent group: White) and inversely associated with age (OR 0.98 [95% CI (0.97-1.0)]. (Table 3) Seropositivity was inversely associated with participants who reported living in homes with multiple age groups (OR 0.33 [95% CI 0.14–0.79], referent group those who live alone. (Table 3)

**Table 3:** Predictors of SARS-CoV-2 positive serology: multivariate analysis

	*Multivariate aOR*	*95% CI*	*P*
**COVID exposure at work**			
No Covid Exposure	REF	—	—
Covid Exposure w/PPE	**3.1**	**1.4–6.9**	**0.007**
Covid Exposure w/out PPE	**4.7**	**2.0–11**	**0.0004**
**Frequency of Physical Contact with Patients**			
Never	REF	—	—
Rarely/Occasionally	1.1	0.33–4.0	0.82
Frequently/Very frequently	1.1	0.29–4.3	0.88
**Frequency of Physical Contact with COVID Patients**			
Never	REF	—	—
Rarely/Occasionally	1.1	0.45–2.8	0.81
Frequently/Very frequently	1.5	0.40–5.2	0.57
**Frequency of working in an Area in Proximity of Patients**			
Never	REF	—	—
Rarely/Occasionally	1.6	0.31–8.1	0.58
Frequently/Very frequently	1.5	0.27–8.3	0.65
**Age group in home**			
Live Alone	REF	—	—
Live with child Age 0–5	0.86	0.19–3.9	0.84
Live with child Age 6–12	0.33	0.02–5.9	0.45
Live with child Age 13–17	0.58	0.03–11.0	0.71
Live with Adult	0.92	0.44–2.0	0.83
Live with Multi-age group in house	**0.33**	**0.14–0.79**	**0.01**
**Frequency of working in an Area in Proximity of COVID Patients**			
Never	REF	—	—
Rarely/Occasionally	1.1	0.42–2.8	0.87
Frequently/Very frequently	1.1	0.34–3.6	0.86
**Frequency of Mask Use in Proximity of Coworkers**			
Never	REF	—	—
Rarely/Occasionally	0.12	0.0–3.1	0.20
Frequently/Very frequently	0.65	0.06–7.6	0.73
**Frequency of Public Mask Use Outside of Work**			
Frequently/Always	REF	—	—
Rarely/Occasionally	2.3	0.80–6.5	0.12
**Never**	**10.1**	**1.9–57**	**0.008**
**Race**			
Asian or Asian American	REF	—	—
Black or African American	1.7	0.51–5.7	0.39
White or Caucasian	1.2	0.52–2.7	0.70
Hispanic/Latino	1.7	0.85–3.4	0.14
Native Hawaiian or other Pacific Islander	**6.3**	**1.6–25**	**0.008**
Other, Decline to state, American Indian or Alaska Native, Mixed Race	0.67	0.31–3.4	0.63
**Age**	**0.98**	**0.97–1.0**	**0.02**
**Do you plan to get the COVID vaccine when available?**			
No/probably no	REF		
Yes/probably yes	0.56	0.3–1.04	0.07
**It is easy for me to do things at work and home to prevent me from getting COVID infection.**			
Agree/strongly agree	REF		
Somewhat disagree/Neither agree or disagree/Somewhat agree	1.0	0.58–1.9	0.89
Strongly disagree/Disagree	0.46	0.09–2.2	0.33
**If I am not careful**,** I will get COVID infection at work.**			
Agree/strongly agree	REF		
Somewhat disagree/Neither agree or disagree/Somewhat agree	0.86	0.38–2.0	0.72
Strongly disagree/Disagree	2.5	0.55–11	0.23
**If I am not careful**,** I will get COVID infection at work.**			
Agree/strongly agree	REF		
Somewhat disagree/Neither agree or disagree/Somewhat agree	0.51	0.21–1.2	0.13
Strongly disagree/Disagree	0.58	0.09–3.6	0.56

Multivariate model includes predictor variables found to significant at the *p* < 0.1 level in bivariate analysis.

## Discussion

We analyzed data from a workplace quality improvement program at a large urban medical center to assess characteristics of persons at risk for SARS-CoV-2 infection. We found a seroprevalence rate of 4.7% for SARS-CoV-2 antibodies among all medical center employees, including both clinical and non-clinical employees. Although we found work and non-work related independent predictors of SARS-CoV-2 seropositivity, interestingly, we found no association between employment type (i.e., clinical versus non-clinical) and seropositivity.

Our results are consistent with some, but not all seropositivity studies performed on healthcare workforce populations [11, 21–23]. For example, our seroprevalence rate of 4.7% is lower than the overall seroprevalence of 8.7% obtained from a meta-analysis [11]. This difference could be explained by different sample populations included in the meta-analysis (global study vs. national study, or rural vs. urban study site), different lockdown and quarantine measures, differences in SARS-CoV-2 antibody tests, and differences in time between when the serology was done compared to the beginning of the pandemic. Similar to Fukuda et al., we found no association between occupation type and SARS-CoV-2 seropositivity [23]. However, Sims et al. found higher seropositivity for specific occupations such as nursing/nursing support, blood sampling, and respiratory therapy [21]. These discordant results could be explained by the differing roles of clinical personnel at different medical centers and differing study designs, among other factors. A broader implication of our study results is seen when comparing healthcare populations that differ by geographic location. For example, Nguyen et al. found that seropositive COVID-19 tests among healthcare workers based in the United Kingdom were greater than a comparable population in the United States (2,747 vs. 1,836 per 100,000 COVID Symptom Study smartphone application users) respectively. These differences may have implications for standardizing global governmental policies surrounding infection control measures during an global pandemic like SARS-CoV-2 [2].

One unique aspect of our study that was not done by other seropositivity surveys on similar medical center populations were the inclusion of questions relating to non-work related SARS-CoV-2 exposures such as community exposure, household co-habitants, and use of infection control behaviors outside the workplace (e.g., masking, hand hygiene, and social distancing) [23, 24]. Inclusion of these questions in our questionnaire allows for richer characterization of our program participants’ lived experience as they relate to potential SARS-CoV-2 exposures because participants do not exclusively dwell solely at their place of work. Additionally, our study was novel in that the program examined attitudes and beliefs about the soon to be released COVID-19 vaccine. The results of our program suggest that exposures, originating from both employment and non-employment/community settings are associated with seropositivity.

SARS-CoV-2 seropositivity was associated with COVID-19 exposure while working at the medical center. Program participants who indicated they had work-related COVID exposures with and, to a greater extent, without PPE use showed a higher likelihood of being seropositive compared to those who indicated no COVID-19 exposure at work. Our results are consistent with a previous COVID-19 seropositivity study assessing the availability of masks and was conducted on healthcare workers pre-COVID-19 vaccine rollout that found PPE availability and presumed use lowered the risk of work-related COVID-19 acquisition [21]. Of note, medical center employees may change their behavior during working hours. For example, they may be adherent to PEE use during patient care, but an oversight in use of PPE at lunch or breaktime [25]. In terms of community SARS-CoV-2 infection, participants who indicated never wearing a mask outside of work had higher odds of seropositivity compared to participants who frequently/always wear a mask outside of work. These results are consistent with previous studies showing the effectiveness of community-based masking in preventing the spread of COVID-19 [26, 27]. Our findings suggest that protection at work from PPE use can easily be negated by lack of PPE use outside the workplace.

Previous studies have shown that minority populations including NHPI race/ethnicity has been disproportionality affected by the SARS-CoV-2 pandemic, including higher case rates and higher mortality [28–30]. Interestingly, despite these observations of racial/ethnic health disparities related to COVID-19 being demonstrated in community settings, our results, which were conducted solely in medical center employees, are consistent with the relationship between seropositivity and NHPI race in more general populations [26]. What drives these racial imbalances are likely multifactorial. For example, strong cultural ties that are characterized by households with a large number of persons and that are multi-generational among this population potentially puts them at higher risk for SARS-CoV-2 exposure [31].

Surprisingly, seropositivity was inversely associated with living at home with multiple age groups. At the time of this program, Los Angeles County based schools were closed for in-class learning, therefore children were home at the time of our program. Los Angeles County health advisories also recommended social distancing (sheltering at home), suggesting program participants’ household members may not have represented a greater risk for SARS-CoV-2 exposure [32, 33]. Our findings suggest that, compared to persons who lived alone, multi-generational households may have practiced greater infection control measures perhaps as a means to protect children, elderly, and generally each other within the same household. Of note, additional household living conditions may also drive COVID-19 infections. For example, poor housing conditions (overcrowding, incomplete kitchen or plumbing facilities), is associated with a 50% higher risk of COVID-19 incidence, however the program’s survey did not collect in depth information on household characteristics [34].

We found no association between seropositivity and program participants attitude and beliefs about COVID-19. These results align with previous findings by our research team showing no association with the Health Belief Model constructs of perceived susceptibility, threat or self-efficacy and vaccine hesitancy [13]. Other phycological determinants of health behavior such as fear, benefit or mistrust may better explain attitudes and beliefs associated with seropositivity that were not assessed in our program.

Our investigation had limitations. First, our seroprevalence program showed low program participation among all medical center employees (∼ 12%) that may contribute to imprecise estimates of seropositivity among medical center employees at our center. It is possible that employees who did not participate in the program had previously gotten tested for SARS-CoV-2 and therefore did not have interest in knowing their prior exposure status offered through the seropositivity program. Alternately, our program may have only attracted persons who were more curious than average about whether they had SARS-CoV-2 infection because they perceived themselves are high risk or were at high risk. This potential selection bias may lower the generalizability of our findings as noted above. A prospective, randomized study addresses selection bias but was not feasible for the current study due to the rapidly changing workplace environment occurring during the COVID-19 pandemic. Nevertheless, our program included diverse employment types such as those with non-patient based care roles including security, administrators, and support staff (i.e., engineering) as well as various allied health professionals such as physical, occupational, and respiratory therapists. Second, our program was unable to determine the source and timing of COVID-19 exposure. However, our questionnaire included questions quantifying participants activities while working (number of physical contacts with patients) and out in the community (frequency of times/week going out in the community, shopping etc.) where potential exposures could be better characterized.

There are strengths to our investigation. First, the sensitivity and specificity of the SARS-CoV-2 IgG antibody test that was used is very high, thereby reducing the chance of underestimating seroprevalence or categorizing uninfected persons as previously infected. Thus, our serologic test minimized the chance of introducing bias. Secondly, our program questionnaire included questions on attitudes and beliefs about a potential COVID-19 vaccine based on constructs present in the Health Belief Model [14, 35]. Use of this model, a robustly studied theory on behavior change, facilitates identification of behavior and health status concordant relationships, and the underlining factors that influence them that may be targets for future disease prevention initiatives [36]. Lastly, our target population was very diverse. It included all employees of medical center and was not limited to employees with patient-based care roles and consisted of a highly diverse race/ethnic employee workforce, thus including persons frequently underrepresented in research studies [37]. To advance health equity, inclusion of minority populations in research studies are essential. Taken together, our program was a robust program designed to better understand SARS-CoV-2 seroprevalence among a high-risk, diverse workforce population in an urban area occurring during time frame with high incidence of COVID-19 infections.

## Conclusion

In summary, our seropositivity program intended to gain a better understanding of the magnitude of prior SARS-CoV-2 infections among its employees that would represent a potential threat to the essential services provided by medical center employees at a large urban medical center. While our findings also support the assertion that higher use of PPE in the workplace is associated with decreased SARS-CoV-2 infection, we also found community exposures were likely a common mechanism for infection acquisition. Therefore, medical centers should consider that workplace infection prevention and control education be expanded, especially during a pandemic, to include greater awareness of the infection risks associated with employee’s behaviors outside of work in addition to the usual messages of protecting oneself during work. Furthermore, given the higher rate of SARS-CoV-2 infection among racial/ethnic groups that may have different beliefs, attitudes, and behaviors in our center, future emergency response and preparedness programs need to incorporate racial and ethnic-specific messages in initiatives to address the health disparities observed during the SARS-CoV-2 pandemic.

## Electronic supplementary material

Below is the link to the electronic supplementary material.

Supplementary Material 1

## Data Availability

The datasets used and/or analyzed during the current study are available from the corresponding author on a reasonable request. Deborah Kupferwasser, dkupferwasser@lundquist.org.

## Abbreviations

Cal/OSHA: California Division of Occupational Safety and Health
COVID-19: Coronavirus Disease of 2019
LAC + USC: Los Angeles County + University of Southern California
NHPI: Native Hawaiian/Pacific Islander
PPE: Personal Protective Equipment
SARS-CoV-2: Severe Acute Respiratory Syndrome Coronavirus 2
