# Supplementary material for: Predictors of seropositivity to SARS-CoV-2 among employees at a large urban medical center
Source: BMC Public Health. 2024 Oct 9;24:2754. doi: 10.1186/s12889-024-20274-6 (PMC11462861; doi:10.1186/s12889-024-20274-6)
Supplement: Supplementary file 1 — Supplementary Material 1 [file 12889_2024_20274_MOESM1_ESM.docx]

# (DHS) QUESTIONAIRE ON COVID ANTIBODY TESTING

**FOR WORKFORCE MEMBERS**

Thank you for having your blood drawn today. DHS is seeking to increase knowledge about ways to prevent spread of COVID-19 in our facilities and the community. To better understand, we are going to ask you some questions. Your answers are very important to us. This survey should take less than 10 minutes to complete.

For this questionnaire we call the coronavirus infection, COVID. For the purpose of this questionnaire, when we say “COVID” we mean the current coronavirus that is causing a worldwide pandemic. COVID is also called “COVID-19”.

**Questionnaire development:**

Response items 1A-1Bii collect information and characterize participant’s potential exposer to COVID-19 infected persons. Questions in this section were based on a previously published survey, however we modified questions for our study to better align with our study goals and specific workforce population. (1)

## 1A. Have ever been exposed to another person with a known COVID infection, such as at work, work outside of DHS facilities, or in a non-work setting such as home or in a public place?

- No 🡪 go to question 1B
- Yes

### 1Ai. (*If yes,*) Where were these exposures? (check *all* that apply)

- As part of my work at DHS
- As part of my work or volunteer activities outside of DHS
- At home
- In the community

### 1Aii. (*If yes,*) Were you wearing proper PPE* during the exposure(s)?

- Yes
- Not always
- No

*PPE = Personal Protective Equipment such as a surgical mask, N95 mask, PAPR, gowns, gloves, face shield or goggles other, as appropriate according to your facility’s policy and procedure.

### 1B. Have you ever had or think you had a COVID infection?

- No 🡪 go to question 2A
- Yes

1Bi. *If yes, check one that best describes your situation*

Did you get tested for COVID **with a swab in your nose or throat**?

- No
- Yes, and one or more tests were **positive**
- Yes, and all tests were **negative**

1Bii. When you had the infection, did you have symptoms of COVID*?

- Yes
- No, I had no symptoms (was asymptomatic)

*COVID symptoms may include cough, shortness of breath, sore throat, fever or chills, loss of sense of taste or smell, headache, muscle or body aches, and diarrhea, etc.

### Next we are going to ask you about your work at the DHS hospitals, clinics, or facilities.

**Questionnaire development:**

Response items in section 2 were developed for this investigation and query about patient contact and number of hours worked at DSH facilities.

**2A. In the past 3 months, *on average* approximately how many hours/week have you been working in DHS hospitals, clinics, or facilities (do not include teleworking from home)?**

hours/week

### 2B. In the past 3 months, *on average,* how often do you have physical contact with patients?

- Never
- Rarely (1-10 physical contacts/day)
- Occasionally (11-20 physical contacts/day)
- Frequently (21-30 physical contacts/day)
- Very frequently (>30 physical contacts/day)

*Physical contact means touching of others with your hands or other parts of your body.

### 2C. In the past 3 months, *on average,* how often do you have physical contact* with patients with known COVID infection?

- Never
- Rarely (1-10 physical contacts/day)
- Occasionally (11-20 physical contacts/day)
- Frequently (21-30 physical contacts/day)
- Very frequently (>30 physical contacts/day)

*Physical contact means touching of others with your hands or other parts of your body.

### 2D. In the past 3 months, *on average,* how often do you work in an area where patients come within 6 feet of you?

- Never
- Rarely (1-25% of the time)
- Occasionally (26-50% of the time)
- Frequently (51-99% of the time)
- Very frequently (100% of the time)

### 2E. In the past 3 months, *on average,* how often do you work in an area where patients with known COVID come within 6 feet of you?

- Never
- Rarely (1-25% of the time)
- Occasionally (26-50% of the time)
- Frequently (51-99% of the time)
- Very frequently (100% of the time)

### Now we are going to ask you about masks. Many people have difficulty wearing masks all of the time. Masks can be uncomfortable, fog up glasses, break, get dirty, etc.

**2F. In the past 3 months, *on average,* during my work at DHS hospitals, clinics, or facilities, how often do you wear a mask within 6 feet of patients?**

- Never
- Rarely (1-25% of the time)
- Occasionally (26-50% of the time)
- Frequently (51-99% of the time)
- Always (100% of the time)
- I don’t interact with patients *and* never come within 6 feet of patients

### Now we are going to ask you about your work duties as they relate to co-workers at DHS hospitals, clinics, or facilities.

**2G. In the past 3 months, *on average,* how often do you have physical contact* with co- workers?**

- Never
- Rarely (1-10 physical contacts/day)
- Occasionally (11-20 physical contacts/day)
- Frequently (21-30 physical contacts/day)
- Very frequently (>30 physical contacts/day)

*Physical contact means touching of others with your hands or other parts of your body.

### 2H. In the past 3 months, *on average,* how often do you work in an area where co-workers come within 6 feet of you?

- Never
- Rarely (1-25% of the time)
- Occasionally (26-50% of the time)
- Frequently (51-99% of the time)
- Very frequently (100% of the time)

### Many people have difficulty wearing masks all of the time. Masks can be uncomfortable, fog up glasses, break, get dirty, etc.

**2I. In the past 3 months, *on average,* during my work at DHS hospitals, clinics, or facilities, how often do you wear a mask within 6 feet of co-workers?**

- Never
- Rarely (1-25% of the time)
- Occasionally (26-50% of the time)
- Frequently (51-99% of the time)
- Always (100% of the time)

### Access to proper hand hygiene* stations or supplies may be difficult. For example, there may be no sinks, inadequate soap supply, or lack of alcohol hand rub.

*Hand hygiene means cleaning your hands with either soap and water or an alcohol hand rub such as a gel or foam.

**2J. In the past 3 months, *on average*, in your work at DHS hospitals, clinics, or facilities, are you able to practice adequate hand hygiene* *when you want to perform hand hygiene*?**

- Never
- Rarely (1-25% of the time)
- Occasionally (26-50% of the time)
- Frequently (51-99% of the time)
- Always (100% of the time)

*Hand hygiene means cleaning your hands with either soap and water or an alcohol hand rub such as a gel or foam.

### Many DHS workforce members have a second part-time job or do volunteer work outside of the DHS hospitals, clinics, or facilities.

**Questionnaire development:**

Response items 3A-3F and 4A-4D were developed for this investigation and query about non-DHS employment, volunteer activities and possible exposures with these activities.

**3A. In the past 3 months, have you worked or volunteered outside of the DHS hospitals, clinics, or facilities?**

- - Yes
  - No 🡪 go to question 4

### 3B. In the past 3 months, *on average*, approximately how many hours/week have you been working or volunteering outside of the DHS hospitals, clinics, and facilities?

hours/week

### Now we are going to ask you about your work or volunteer activities outside of the DHS hospitals, clinics, or facilities

**3C. In the past 3 months, *on average,* in your work or volunteer activities outside of the DHS hospitals, clinics, or facilities how often do you have physical contact* with other people?**

- Never
- Rarely (1-10 physical contacts/day)
- Occasionally (11-20 physical contacts/day)
- Frequently (21-30 physical contacts/day)
- Very frequently (>30 physical contacts/day)

*Physical contact means touching of others with your hands or other parts of your body.

### 3D. In the past 3 months, *on average,* in your work or volunteer activities outside of the DHS hospitals, clinics, or facilities, how often do other people come within 6 feet of you?

- Never
- Rarely (1-25% of the time)
- Occasionally (26-50% of the time)
- Frequently (51-99% of the time)
- Very frequently (100% of the time)

### Many people have difficulty wearing masks all of the time. Masks can be uncomfortable, fog up glasses, break, get dirty, etc.

**3E. In the past 3 months, *on average*, in your work or volunteer activities outside of the DHS hospitals, clinics, or facilities, how often do you wear a mask within 6 feet of others?**

- Never
- Rarely (1-25% of the time)
- Occasionally (26-50% of the time)
- Frequently (51-99% of the time)
- Always (100% of the time)
- I don’t interact with people *and* never come within 6 feet of patients as part of this work

### Access to proper hand hygiene* stations or supplies may be difficult. For example, there may be no sinks, inadequate soap supply, or lack of alcohol hand rub.

*Hand hygiene means cleaning your hands with either soap and water or an alcohol hand rub such as a gel or foam.

### 3F. In the past 3 months, *on average*, in your work or volunteer activities outside of the DHS hospitals, clinics, or facilities, how often are you able to practice adequate hand hygiene* when you want to perform hand hygiene?

- Never
- Rarely (1-25% of the time)
- Occasionally (26-50% of the time)
- Frequently (51-99% of the time)
- Always (100% of the time)

*Hand hygiene means cleaning your hands with either soap and water or an alcohol hand rub such as a gel or foam.

## EXPOSURE OUTSIDE OF WORK

### Now we are going to ask you some questions about possible COVID exposures outside of work.

4A. Other than yourself, how many people live in your home?

- - 0 🡪 go to question 4B
  - 1 or more

4Ai. Of these people, how many are: Children age 0-5

Children age 6-12

Children age 13-17 Adult age 18-100

4B. In the past 3 months, *on average*, how often to you typically go shopping for groceries or do other errands:

- - Never
  - 1-2 times/week
  - 3-6 times a week
  - 7 times a week or more

4C. In the past 3 months, *on average*, when you are **not at work or volunteer activities**, how often do you wear a **mask** of any kind **in public** when you are less than 6 feet from other persons:

- - Never
  - Rarely (1-25% of the time)
  - Occasionally (26-50% of the time)
  - Frequently (51-99% of the time)
  - Always (100% of the time)

4D. In the past 3 months, *on average*, when you are **not at work or volunteer activities**, how often are you able to practice adequate **hand hygiene*** when you want to perform hand hygiene?

- - Never
  - Rarely (1-25% of the time)
  - Occasionally (26-50% of the time)
  - Frequently (51-99% of the time)
  - Always (100% of the time)

*Hand hygiene means cleaning your hands with either soap and water or an alcohol hand rub such as a gel or foam.

## ATTITUDES TOWARD VACCINATION

### Many experts think a vaccine for COVID will be available in 2021. We don’t know if this will happen. Assuming that a vaccine for COVID is available next year, we would like to better understand your feelings towards a COVID vaccine.

**Questionnaire development description:**

### Questions 5A-5D were developed based on a published study published study (1), and queried about attitudes toward a COVID-19 vaccine.

5A. If your test shows you are NOT immune to COVID, do you plan to get the COVID vaccine when available?

- - Yes
  - Probably yes
  - Probably no
  - No

5B. which of the following would be reasons why you would get a COVID vaccine? (Select *all* that apply)

- - Government leaders recommend I get vaccinated
  - My healthcare provider recommends I get vaccinated
  - It would allow for life to go back to normal if most people are vaccinated
  - I want to protect my community
  - I want to protect my family
  - I want to protect myself

5C. Which of the following would be reasons why you would NOT get a COVID vaccine? (Select ***all*** that apply)

- - I am healthy and do not need to be immunized
  - I am worried about vaccine side effects in general
  - I am worried about vaccine side effects of a COVID vaccine
  - I am worried the COVID vaccine will cause me to have a COVID infection
  - I do not think the COVID vaccine will work very well
  - I do not think the COVID pandemic is as serious as people say it is
  - I do not like needles
  - I do not have time to get vaccinated
  - Unless DHS pays for it, I cannot pay for the COVID vaccine

5D. Each year, do you get an annual flu (influenza) vaccine?

- - Never
  - Sometimes
  - Always

## PERSONNEL INFORMATION:

**Questionnaire development:**

Section 6 of our questionnaire collects demographic information.

6A. Which best describes your job description?

- - Administrative / Office Staff **□** Dietary staff
  - Security **□** Environmental Services
  - Physician **□** Nurse
  - Pharmacist **□** Respiratory therapy
  - Phlebotomist **□** Physical Therapy/Occupational Therapy
  - Facilities management **□** Technician, patient care related
  - Social worker/case manager **□** Laboratory staff
  - Other, please state

6B. What is your *primary* work site?

- - LAC+USC
  - Harbor-UCLA
  - Olive View-UCLA
  - Rancho Los Amigos
  - MLK
  - High Desert
  - Ambulatory Care Clinic
  - Other, please state

6C. What is your current gender:

- - Male
  - Female
  - Transgender

6D. Age: years

6E. Race:

- - American Indian / Alaskan Native **□** African American / Black
  - Asian **□** Hispanic or Latino
  - White **□** Native Hawaiian / Pacific Islander
  - Mixed race **□** Other
  - Decline to state

### You are almost done with the survey. We value and want to hear your opinions about COVID.

**Questionnaire development:**

Section 7 of our questionnaire contains response items developed based on the Health Belief Model psychological constructs of: perceived susceptibility (7A, 7G and 7H), perceived threat (7B, and 7I), and perceived self-efficacy (7C, 7D, 7E, 7F, and 7J), as they relate to the COVID-19 pandemic. (2, 3)

**7A. I am worried about being infected by COVID.**

- - Strongly disagree
  - Disagree
  - Somewhat disagree
  - Neither agree or disagree
  - Somewhat agree
  - Agree
  - Strongly agree

### 7B. COVID affects me emotionally, such as making me feel furious, afraid, angry, or depressed.

- - Strongly disagree
  - Disagree
  - Somewhat disagree
  - Neither agree or disagree
  - Somewhat agree
  - Agree
  - Strongly agree

### 7C. Wearing a mask is important to protect me from COVID.

- - Strongly disagree
  - Disagree
  - Somewhat disagree
  - Neither agree or disagree
  - Somewhat agree
  - Agree
  - Strongly agree

### 7D. Frequent hand hygiene* is important to protect me from getting COVID

- - Strongly disagree
  - Disagree
  - Somewhat disagree
  - Neither agree or disagree
  - Somewhat agree
  - Agree
  - Strongly agree

*Hand hygiene means cleaning your hands with either soap and water or an alcohol hand rub such as a gel or foam.

### 7E. Outside of my home, keeping more than 6 feet away from others is important to protect me from getting COVID infection.

- - Strongly disagree
  - Disagree
  - Somewhat disagree
  - Neither agree or disagree
  - Somewhat agree
  - Agree
  - Strongly agree

### 7F. It is easy for me to do things at work and home to prevent me from getting COVID infection

- - Strongly disagree
  - Disagree
  - Somewhat disagree
  - Neither agree or disagree
  - Somewhat agree
  - Agree
  - Strongly agree

### 7G. If I am NOT careful, I will get COVID infection at work.

- - Strongly disagree
  - Disagree
  - Somewhat disagree
  - Neither agree or disagree
  - Somewhat agree
  - Agree
  - Strongly agree

### 7H. If I am NOT careful, I will get COVID infection while in the community doing activities such as shopping, interacting with others.

- - Strongly disagree
  - Disagree
  - Somewhat disagree
  - Neither agree or disagree
  - Somewhat agree
  - Agree
  - Strongly agree

### 7I. If I get COVID infection, I will get severely ill and may need to be hospitalized.

- - Strongly disagree
  - Disagree
  - Somewhat disagree
  - Neither agree or disagree
  - Somewhat agree
  - Agree
  - Strongly agree

### 7J. People around me at work at DHS facilities are routinely wearing masks when they are less than 6 feet away from me and others.

- - Strongly disagree
  - Disagree
  - Somewhat disagree
  - Neither agree or disagree
  - Somewhat agree
  - Agree
  - Strongly agree

*Hand hygiene means cleaning your hands with either soap and water or an alcohol hand rub such as a gel or foam.

# End of survey. Thank you for your time and effort.

# REFERENCES

1. Fisher KA, Bloomstone SJ, Walder J, Crawford S, Fouayzi H, Mazor KM. Attitudes Toward a Potential SARS-CoV-2 Vaccine : A Survey of U.S. Adults. Ann Intern Med. 2020;173(12):964-73.

2. Wong LP, Alias H, Wong PF, Lee HY, AbuBakar S. The use of the health belief model to assess predictors of intent to receive the COVID-19 vaccine and willingness to pay. Hum Vaccin Immunother. 2020;16(9):2204-14.

3. Fall E, Izaute M, Chakroun-Baggioni N. How can the health belief model and self-determination theory predict both influenza vaccination and vaccination intention ? A longitudinal study among university students. Psychol Health. 2018;33(6):746-64.
